# Changing Interdigestive Migrating Motor Complex in Rats under Acute Liver Injury

**DOI:** 10.1155/2014/634281

**Published:** 2014-10-28

**Authors:** Mei Liu, Su-Jun Zheng, Weihong Xu, Jianying Zhang, Yu Chen, Zhongping Duan

**Affiliations:** ^1^Artificial Liver Center, Beijing You'an Hospital, Capital Medical University, Beijing 100069, China; ^2^Department of Biological Sciences, The University of Texas at El Paso, El Paso, TX 79968, USA; ^3^Department of Rheumatology, Second Affiliated Hospital, School of Medicine, Zhejiang University, Hangzhou 310009, China

## Abstract

Gastrointestinal motility disorder is a major clinical manifestation of acute liver injury, and interdigestive migrating motor complex (MMC) is an important indicator. We investigated the changes and characteristics of MMC in rats with acute liver injury. Acute liver injury was created by d-galactosamine, and we recorded the interdigestive MMC using a multichannel physiological recorder and compared the indexes of interdigestive MMC. Compared with normal controls, antral MMC Phase I duration was significantly prolonged and MMC Phase III duration was significantly shortened in the rats with acute liver injury. The duodenal MMC cycle and MMC Phases I and IV duration were significantly prolonged and MMC Phase III duration was significantly shortened in the rats with acute liver injury. The jejunal MMC cycle and MMC Phases I and IV duration were significantly prolonged and MMC Phase III duration was significantly shortened in the rats with acute liver injury compared with normal controls. Compared with the normal controls, rats with acute liver injury had a significantly prolonged interdigestive MMC cycle, related mainly to longer MMC Phases I and IV, shortened MMC Phase III, and MMC Phase II characterized by increased migrating clustered contractions, which were probably major contributors to the gastrointestinal motility disorders.

## 1. Introduction

The liver is the largest digestive gland in the human body and plays an important role in detoxification, secretion, synthesis, and conversion of lots of substances in human body. When the hepatic tissue is injured, other organs of the body are all affected to varying degrees, among which the gastrointestinal tract is the earliest and most seriously affected one. Interdigestive migrating motor complex (MMC) is a pattern of cyclic motor activity of the stomach and small intestine during digestion [1]. It consists of periods of inactivity alternating with segmental or propulsive contractions and generally originates in the gastric antrum or duodenum, spreads abroad, and reaches as far as the colon. Abnormal MMC frequency and intensity may induce abdominal distention, early satiety, anorexia, abdominal pain, constipation, and other gastrointestinal abnormalities, which are often seen in patients with liver injury. However, there are few studies on the relation between liver injury and MMC.

In the case of acute liver injury, the intestinal mucosa is damaged, and consequently intestinal flora, intestinal permeability, the interstitial cells of Cajal (ICCs), intestinal neurons, and motilin secretion become abnormal. As a result, the intestinal MMC changes and a series of clinical symptoms of acute liver injury may appear. The gastrointestinal tract is the earliest and most seriously affected organ in addition to the liver. Disorders of gastrointestinal motility affect recovery from liver injury and even intensify liver damage. Gastrointestinal motility disorder is one of the main indicators of hepatic gastrointestinal dysfunction, and the MMC motor pattern is an important indicator of gastrointestinal motility disorder.

Thus, we reasoned that changes in MMC may occur in acute liver injury, leading to gastrointestinal clinical manifestations. In this study, we tried to detect MMC changes during acute liver injury, which is essential to clarify the mechanism of hepatic gastrointestinal dysfunction. Finally, we observed the changes in interdigestive MMC of rats with acute liver injury. In the preliminary stage, our findings showed the relationship between acute liver injury and MMC, which may provide clues for feasible clinical treatments of gastrointestinal symptoms in patients with liver injury.

## 2. Materials and Methods

### 2.1. Experimental Animal

Thirty purebred Sprague-Dawley rats of either sex (pathogen-free, weighing 220–250 g; Animal Center of Chinese Academy of Military Sciences, Beijing, China) were used. They were randomly divided into two groups: 15 in the normal control group and 15 in the acute liver injury group. During the experiment, two rats in the acute liver injury group and one in the normal control group died. Anatomical analysis confirmed that they died of intestinal obstruction. This study was carried out in strict accordance with the recommendations in the Guide for the Care and Use of Laboratory Animals of Capital Medical University, Beijing, China. The protocol was approved by the Committee on the Ethics of Animal Experiments of Capital Medical University, Beijing, China. All surgeries were performed under chloral hydrate anesthesia, and all efforts were made to minimize suffering.

### 2.2. Drugs and Materials

We purchased d-galactosamine from Jiangsu Qidong Jiufeng Industry & Trade Co. Ltd., Qidong city, China, PTFE film wrapped wire from Shanghai Special Cable Company, Shanghai, China, and MP36 multichannel physiological recorder from BIOPAC Systems, Santa Barbara, CA, USA.

### 2.3. Electrode Implantation

Rats were fasted for 8 h and intraperitoneally anaesthetized with chloral hydrate (0.4 g/kg) prior to the experiment. The hair of the abdomen was shaved. A longitudinal incision was made aseptically along the median line of the stomach. One pair of silver electrodes was implanted in the seromuscular layer of the antrum, duodenum, and jejunum, respectively, tunneled subcutaneously, and exteriorized and fixed at the nape of the neck. The abdominal cavity was given penicillin sodium to resist infection, and then the incision was secured. Glucose in normal saline was given to complement the loss of blood during the operation. After fasting for 1 day without water deprivation after the operation, rats were housed individually and cleanly for 3-day recovery, during which they were allowed to drink water and eat food freely. After that, rats underwent electrophysiological experiments.

### 2.4. Preparing Acute Liver Injury Models

Without fasting prior to the experiment, rats were given 1.2 g/kg d-galactosamine by intraperitoneal injection (d-galactosamine in sterile normal saline, with 1 mol/L sodium hydroxide to adjust pH to ~7.0 and concentration to 0.5 g/mL) to induce acute liver injury. Rats in the normal control group were injected intraperitoneally with the same amount of normal saline. Within 48–72 h after drug or saline administration, we removed liver specimens for pathological examination and confirmed hepatic pathological changes.

### 2.5. Monitoring Gastrointestinal MMC

Rats were fasted for 18–24 h but were allowed free access to water prior to the experiment. In the conscious state, they were fixed to the stabilizing devices. For recording gastrointestinal MMC, three pairs of silver electrodes from the dorsal surface of the skin were connected with an MP36 multichannel physiological recorder. Rats in the normal control group, within 48–72 h after intraperitoneal injection of normal saline, were recorded for ~2 h, and at least three MMC cycles from each rat were obtained. Rats in the acute liver injury group, within 48–72 h after d-galactosamine administration, were recorded in the same way for ~3 h, and at least three MMC cycles were obtained from each rat.

### 2.6. Dividing Four Cyclic Phases of MMC

MMC was divided into four different phases according to the number of starting potentials loaded with action potentials and the amplitude of action potentials. In Phase I each starting potential was unloaded with or occasionally had an action potential; the starting potentials loaded with action potentials accounted for no more than 5%. In Phase II the starting potentials with irregular action potentials after Phase I accounted for >5% but <95% of the total starting potentials. Phase III was characterized by a sudden burst and sudden stop, and continuous and high-amplitude action potential bursts at almost every starting potential; therefore, the starting potentials loaded with action potentials accounted for >95% of the total starting potentials. At Phase IV, spike potential activity and contraction activity rapidly declined, but the starting potentials loaded with action potentials still accounted for >5% of the total starting potentials. Phase IV was a transition period after Phase III and before Phase I.

### 2.7. Statistical Analysis

Results were expressed as mean ± SD. SPSS version 17.0 software was used for the statistical analysis. The* t*-test of two independent samples was conducted for the data in line with normal distribution and data with homogeneity of variance. The Mann-Whitney *U* test was conducted for the data with heterogeneity of variance. *P* < 0.05 was considered statistically significant.

## 3. Results

### 3.1. Acute Liver Injury and MMC

The acute liver injury group of rats was characterized by reduced activity, malaise, poor appetite, and reduced water drinking at 12 h after d-galactosamine administration. As liver failure progressed, the rats were generally lethargic, somnolent, and unresponsive, and water drinking further decreased at 24–48 h after d-galactosamine administration. At 72 h after d-galactosamine administration, the rats gradually resumed activities, which basically returned to normal at 2 weeks after d-galactosamine administration.

**Figure 1:**
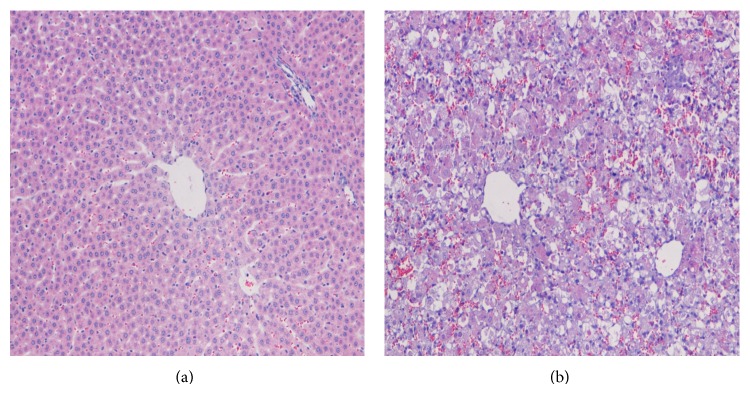
Representative H&E-stained liver tissue sections of rats that were injected with d-galactosamine (1.2 g/kg intraperitoneally) for induction of acute liver injury. (a) Sham-operated animals without liver injury served as controls. (b) Liver tissue from rats with acute liver injury exhibited disruption of the general architecture and microvascular disintegration, as well as tissue apoptosis and necrosis. Morphological changes, such as vacuolization, swollen cytoplasm with disrupted cell, and organelle membranes, as well as lytic nuclear changes served to determine necrosis. H&E: hematoxylin and eosin.

Histological analysis showed that the control livers were characterized by normal hepatic configuration, ruddy color, fine surface grain, slightly tough texture, normal hepatic structure, clear hepatic lobule structure, and no degeneration or necrosis of hepatic cells. At 60 h after administration of d-galactosamine, the livers of rats with acute liver injury showed marked albuminous swelling, mild steatosis, punctate necrosis of hepatic cells, bile duct hyperplasia of the portal area, and expansion of the central vein and hepatic sinusoids, as well as lymphocytic infiltration (Figure 1).

We then examined MMC in acute liver injury. Phase III is a high-amplitude contraction period. Among the four phases, MMC Phase III has important physiological significance: with the intensification of gastrointestinal motility, the contraction wave in this phase accelerates transmission and acts as a “street sweeper” in the gastrointestinal tract. Phase I is an inactivity relaxation period; Phase II is an irregular contraction period; and Phase IV is a contraction regression period—a short transition period after Phase III and before Phase I. The mechanism of MMC may be closely related to serum motilin level, ICCs, or the cholinergic nerves of the enteric nervous system (ENS), which may change in liver injury. The results of this study were analyzed by statistical software. Compared with normal controls, the antral MMC Phase I duration was significantly prolonged (*P* < 0.05). There were no significant differences between the two groups in MMC cycle and MMC Phases II and IV duration (*P* > 0.05). MMC Phase III duration was significantly shortened (*P* < 0.05) in the rats with acute liver injury. Duodenal MMC Phase II duration did not change significantly (*P* > 0.05), while MMC cycle and duration of MMC Phases I and IV were significantly prolonged (*P* < 0.05) and MMC Phase III duration was significantly shortened (*P* < 0.05) in the rats with acute liver injury. The jejunal MMC Phase II duration did not change significantly (*P* > 0.05), while MMC cycle and duration of MMC Phases I and IV were significantly prolonged (*P* < 0.05) and MMC Phase III duration was significantly shortened (*P* < 0.05) in the rats with acute liver injury (Tables 1, 2, and 3).

### 3.2. MMC Waveform Characteristics in Acute Liver Injury

**Figure 2:**
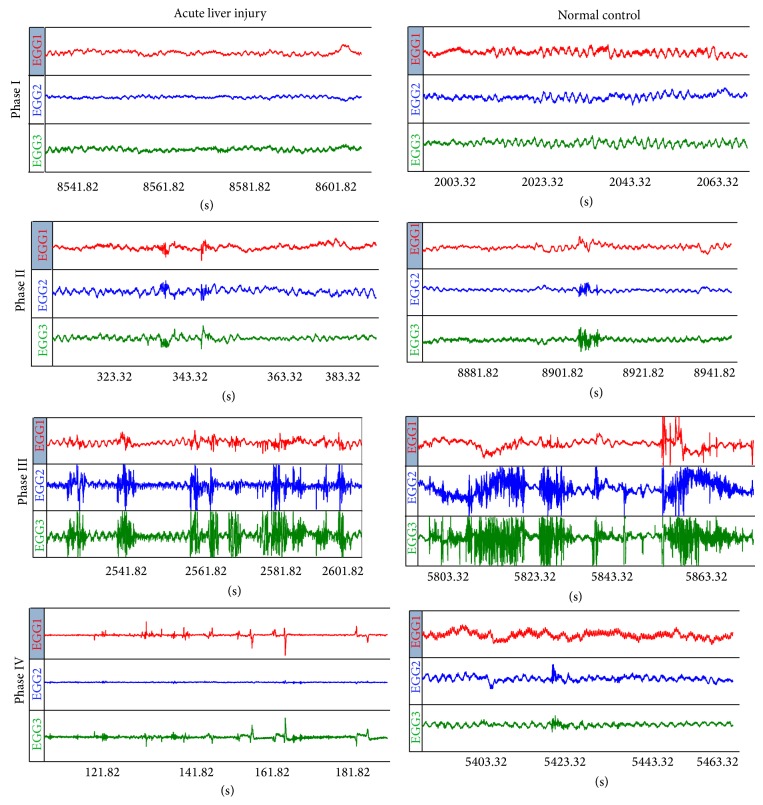
MMC phases of normal rats and rats with acute liver injury. MMC of rats with acute liver injury is characterized by insignificant changes in MMC Phase II duration but increased migrating clustered contractions of MMC Phase II, significantly shortened MMC Phase III duration, and decreased amplitude and irregular shape of MMC Phase III. The red, blue, and green colors of MMC Phase III came from the gastric antrum, duodenum, and jejunum, respectively.

Compared with the normal control group, the acute liver injury group was characterized by significantly prolonged interdigestive MMC cycles, related in large part to longer MMC Phases I and IV, insignificant changes in MMC Phase II duration, but increased migrating clustered contractions of MMC Phase II, significantly shortened MMC Phase III duration, and decreased amplitude and irregular shape of MMC Phase III (Figure 2).

## 4. Discussion

The current study has shown that, compared with the normal control group, the acute liver injury group is characterized by three features: a significantly prolonged interdigestive MMC cycle, related in large part to longer MMC Phases I and IV; increased migrating clustered contractions of MMC Phase II; and significantly shortened MMC Phase III and decreased amplitude and irregular shape of MMC Phase III, which are different from the characteristics of MMC changes in liver cirrhosis. In addition, as a result of prolongation of the inactivity period of MMC Phases I and IV and the significant shortening of MMC Phase III, the intensive stomach contractions become weakened, and the stomach contents cannot be propelled to the duodenum in the absence of a powerful drive. Similarly, when Phase III duration is significantly shortened, the strong contractions of the small intestine are also weakened. As a result, various residual substances remain in the small intestine and cannot be transported to the colon and excreted from the body. It is unknown whether the above findings contribute to the occurrence of abdominal distention, early satiety, anorexia, abdominal pain, constipation, and other gastrointestinal abnormalities of patients with acute liver injury.

The underlying mechanism for MMC remains controversial. Studies have shown that MMC changes may be closely related to motilin. The contractions of MMC Phase III are related to the peak value of periodic fluctuations of plasma motilin [2]. Intravenous administration of physiological doses of motilin can stimulate the occurrence of MMC in the gastrointestinal tract [3], while the application of low-dose erythromycin (motilin receptor agonist) can also induce the occurrence of MMC Phase III in the gastric antrum. Therefore, most scholars believe that MMC may be induced by motilin. However, MMC may not necessarily appear when the motilin concentration reaches its peak value in the blood, which means that MMC may be regulated by other mechanisms [4]. The ICCs are pacemaker cells of the gastrointestinal slow wave, and the patch clamp technique has confirmed that ICCs are a generator of gastrointestinal slow waves and are closely related to the occurrence of MMC [5]. However, Spencer et al. pointed out that MMC occurs even in W/W^v^ mutant mice in the absence of both ICCs and slow waves of the jejunal myenteron, which indicates that MMC is not only controlled by ICCs, but also possibly regulated by the ENS [6]. The study of Matsufuji and Yokoyama showed that MMC can be interrupted by hexamethonium and tetrodotoxin and intensified by apamin and suramin, which means that MMC may be regulated by the cholinergic nerves of the ENS [7]. Another study showed that the vagus nerve is essential for initiating postprandial contractions, and inhibition of the Phase III-like contractions induced by motilin is highly dependent on the vagus nerve [8].

The latest studies have shown that bile secretion and excretion can affect the peak current amplitude and duration of MMC [9], and the MMC cycle is significantly shortened after subtotal hepatectomy [10]. Changes in intestinal flora can affect the progression of acute liver injury, and flora balance is beneficial to the recovery from liver injury and alleviation of abdominal distention, diarrhea, and other symptoms [11]. Abell et al. have found that the majority of patients with chronic nausea and vomiting have remarkably abnormal gastrointestinal current and ENS [12]. The electric field of MMC formed in the gastrointestinal tract can help lactobacilli and other probiotics to be adsorbed onto the epithelium of the intestinal mucosa, to improve their colonization environment, and to enhance their competitiveness against Gram-negative bacteria [13]. It has been shown that the mucosa is not required for MMC generation since removal of the mucosa and blockade of serotonin release from enterochromaffin cells (EC cells) do not prevent MMCs. The clinical symptoms of acute liver injury are closely related to intestinal flora, gastrointestinal MMC, and the ENS, and both sides interact with and influence each other.

The mechanism by which acute liver injury modifies MMCs is unclear but is unlikely to be mediated via the mucosa, since the mucosa is not required for MMC generation. The mechanism that generates MMCs is localized to the myenteric plexus and/or muscularis externa and occurs independently of the mucosa. MMC changes during acute liver injury have rarely been reported thus far. Studies have shown that cirrhosis can cause changes in gastrointestinal motility, which may be attributed to MMC changes [14–16]. According to Madrid et al., patients with liver cirrhosis have a prolonged MMC cycle, related in large part to a longer Phase II. Migrating cluster contractions occur often at MMC Phase II, and as a result the frequency of MMC Phase III is reduced and the clearance function of the small intestine is affected [17]. Haisheng et al. had similar results [18]. Liu et al. found significant changes in MMC Phases II and III in rats with acute liver failure [19].

The present study only showed the changes and characteristics of MMC in rats with acute liver injury, and the mechanism of these changes needs further study, based on a large number of basic and clinical experiments. Changes in concentration of various gastrointestinal hormones, amount of relevant hormone receptors, and intestinal permeability and gastrointestinal flora during acute liver injury may be helpful in the discussion of the mechanism of MMC changes in acute liver injury.

We found that MMC had significant changes during acute liver injury. One possibility why MMC was altered was because of inflammation of the bowel and disruption of the pacemaker mechanism that generates MMCs, which has been shown to lie in the myenteric plexus and/or muscularis externa. In the case of acute liver injury, intestinal flora, intestinal permeability, ICCs, intestinal neurons, and motilin secretion become abnormal. As a result, the intestinal MMC changes and a series of clinical symptoms of acute liver injury may appear. The gastrointestinal tract is the earliest and most seriously affected organ, except the liver, in the course of liver failure. The more severe the liver disease, the more intense the gastrointestinal dysfunction, and gastrointestinal dysfunction affects recovery from liver injury and even intensifies the liver damage. Gastrointestinal motility disorder is one of the main indicators of hepatic gastrointestinal dysfunction, and MMC is an important indicator that reflects gastrointestinal motility disorder. The study of MMC changes during acute liver injury can help to clarify the mechanism of hepatic gastrointestinal dysfunction.

## Figures and Tables

**Table 1:** Comparison of antral MMC in rats with acute liver injury and normal control group ($\bar{X}\pm S$).

MMC phase	Acute liver injury group (*n* = 13)	Normal control group (*n* = 14)	*t* or *U* value	*P* value
Phase I	677.5 ± 280.62^★^	442.67 ± 223.38	*t* = −2.73	0.01
Phase II	558.00 ± 271.52	569.75 ± 320.19	*t* = 0.11	0.91
Phase III	37.57 ± 9.96^★^	60.56 ± 22.35	*t* = 3.64	<0.01
Phase IV	313.00 ± 262.36	165.36 ± 59.99	*U* = −1.93	0.08
MMC cycle	1566.31 ± 389.50	1336.04 ± 545.90	*t* = −1.35	0.19

^★^ *P* < 0.05 versus normal control group.

**Table 2:** Comparison of duodenal MMC in rats with acute liver injury and normal control group ($\bar{X}\pm S$).

MMC phase	Acute liver injury group (*n* = 13)	Normal control group (*n* = 14)	*t* or *U* value	*P* value
Phase I	734.94 ± 208.30^★^	455.54 ± 233.89	*t* = −3.86	<0.01
Phase II	577.44 ± 379.86	576.00 ± 323.06	*t* = −0.01	0.99
Phase III	36.16 ± 11.33^★^	61.36 ± 21.84	*t* = 4.58	<0.01
Phase IV	381.84 ± 197.89^★^	166.68 ± 64.30	*U* = −4.56	<0.01
MMC cycle	1716.25 ± 397.50^★^	1328.24 ± 535.70	*t* = −2.49	0.02

^★^ *P* < 0.05 versus normal control group.

**Table 3:** Comparison of jejunal MMC in rats with acute liver injury and normal control group ($\bar{X}\pm S$).

MMC phase	Acute liver injury group (*n* = 13)	Normal control group (*n* = 14)	*t* or *U* value	*P* value
Phase I	741.75 ± 222.72^★^	453.63 ± 248.62	*t* = −3.74	<0.01
Phase II	582.44 ± 391.72	612.25 ± 386.66	*t* = 0.24	0.81
Phase III	36.16 ± 11.33^★^	59.80 ± 18.59	*t* = 4.89	<0.01
Phase IV	380.84 ± 217.32^★^	165.48 ± 71.33	*U* = −4.15	<0.01
MMC cycle	1716.38 ± 400.04^★^	1320.04 ± 504.22	*t* = −2.65	0.01

^★^ *P* < 0.05 versus normal control group.
